# Evolution of anelloviruses from a circovirus-like ancestor through gradual augmentation of the jelly-roll capsid protein

**DOI:** 10.1093/ve/vead035

**Published:** 2023-05-27

**Authors:** Anamarija Butkovic, Simona Kraberger, Zoe Smeele, Darren P Martin, Kara Schmidlin, Rafaela S Fontenele, Michelle R Shero, Roxanne S Beltran, Amy L Kirkham, Maketalena Aleamotu’a, Jennifer M Burns, Eugene V Koonin, Arvind Varsani, Mart Krupovic

**Affiliations:** Institut Pasteur, Université Paris Cité, CNRS UMR6047, Archaeal Virology Unit, 25 rue du Dr Roux, Paris 75015, France; The Biodesign Center for Fundamental and Applied Microbiomics, Center for Evolution and Medicine, School of Life Sciences, Arizona State University, 1001 S. McAllister Ave, Tempe, AZ 85287, USA; The Biodesign Center for Fundamental and Applied Microbiomics, Center for Evolution and Medicine, School of Life Sciences, Arizona State University, 1001 S. McAllister Ave, Tempe, AZ 85287, USA; The Biodesign Center for Fundamental and Applied Microbiomics, Center for Evolution and Medicine, School of Life Sciences, Arizona State University, 1001 S. McAllister Ave, Tempe, AZ 85287, USA; The Biodesign Center for Fundamental and Applied Microbiomics, Center for Evolution and Medicine, School of Life Sciences, Arizona State University, 1001 S. McAllister Ave, Tempe, AZ 85287, USA; The Biodesign Center for Fundamental and Applied Microbiomics, Center for Evolution and Medicine, School of Life Sciences, Arizona State University, 1001 S. McAllister Ave, Tempe, AZ 85287, USA; Biology Department, Woods Hole Oceanographic Institution, 266 Woods Hole Rd, Woods Hole, MA 02543, USA; Department of Ecology and Evolutionary Biology, University of California Santa Cruz, 130 McAllister Way, Santa Cruz, CA 95060, USA; U.S. Fish and Wildlife Service, Marine Mammals Management, 1011 E, Tudor Road, Anchorage, AK 99503, USA; School of Environmental and Life Sciences, The University of Newcastle, University Drive, Callaghan, NSW 2308, Australia; Department of Biological Sciences, Texas Tech University, 2500 Broadway, Lubbock, TX 79409, USA; National Center for Biotechnology Information, National Library of Medicine, 8600 Rockville Pike, Bethesda, MD 20894, USA; The Biodesign Center for Fundamental and Applied Microbiomics, Center for Evolution and Medicine, School of Life Sciences, Arizona State University, 1001 S. McAllister Ave, Tempe, AZ 85287, USA; Computational Biology Division, Department of Integrative Biomedical Sciences, Institute of Infectious Diseases and Molecular Medicine, University of Cape Town, Observatory, 1 Anzio Road, Cape Town 7925, South Africa; Institut Pasteur, Université Paris Cité, CNRS UMR6047, Archaeal Virology Unit, 25 rue du Dr Roux, Paris 75015, France

**Keywords:** Anellovirus, structural modelling, capsid proteins, jelly-roll fold, virus evolution, taxonomy and classification, *Commensaviricota*

## Abstract

Anelloviruses are highly prevalent in diverse mammals, including humans, but so far have not been linked to any disease and are considered to be part of the ‘healthy virome’. These viruses have small circular single-stranded DNA (ssDNA) genomes and encode several proteins with no detectable sequence similarity to proteins of other known viruses. Thus, anelloviruses are the only family of eukaryotic ssDNA viruses currently not included in the realm *Monodnaviria*. To gain insights into the provenance of these enigmatic viruses, we sequenced more than 250 complete genomes of anelloviruses from nasal and vaginal swab samples of Weddell seal (*Leptonychotes weddellii*) from Antarctica and a fecal sample of grizzly bear (*Ursus arctos horribilis*) from the USA and performed a comprehensive family-wide analysis of the signature anellovirus protein ORF1. Using state-of-the-art remote sequence similarity detection approaches and structural modeling with AlphaFold2, we show that ORF1 orthologs from all *Anelloviridae* genera adopt a jelly-roll fold typical of viral capsid proteins (CPs), establishing an evolutionary link to other eukaryotic ssDNA viruses, specifically, circoviruses. However, unlike CPs of other ssDNA viruses, ORF1 encoded by anelloviruses from different genera display remarkable variation in size, due to insertions into the jelly-roll domain. In particular, the insertion between β-strands H and I forms a projection domain predicted to face away from the capsid surface and function at the interface of virus–host interactions. Consistent with this prediction and supported by recent experimental evidence, the outermost region of the projection domain is a mutational hotspot, where rapid evolution was likely precipitated by the host immune system. Collectively, our findings further expand the known diversity of anelloviruses and explain how anellovirus ORF1 proteins likely diverged from canonical jelly-roll CPs through gradual augmentation of the projection domain. We suggest assigning *Anelloviridae* to a new phylum, ‘*Commensaviricota*’, and including it into the kingdom *Shotokuvirae* (realm *Monodnaviria*), alongside *Cressdnaviricota* and *Cossaviricota*.

## Introduction

Anelloviruses (family *Anelloviridae*) are one of the most enigmatic components of the human virome, in terms of both their biology and provenance. A large proportion of the human population appears to be infected with anelloviruses, yet no disease has been unequivocally linked to anellovirus infections (Kaczorowska and van der Hoek 2020; Webb, Rakibuzzaman, and Ramamoorthy 2020). The infections in humans have been shown to occur at an early age (Kaczorowska et al. 2022a), with high virus loads detected throughout the lifetime, and have been identified in nearly every human tissue (Leppik et al. 2007; Focosi et al. 2016; Blatter et al. 2018) including the brain and in fluid samples (Gerner et al. 2000; Goto et al. 2000; Inami et al. 2000; Itoh et al. 2000; Maggi et al. 2001; Naganuma et al. 2008; Arze et al. 2021; Kaczorowska et al. 2022b, 2023), consistent with the lymphocytes being the primary site of anellovirus replication (Leppik et al. 2007). The virus load appears to be controlled by the immune system because virus levels increase with the level of host immunosuppression (Focosi et al. 2016; Blatter et al. 2018). Although various studies have indicated a possible association between the presence of anelloviruses and certain medical conditions, such as diabetes, fever, cancer, liver cirrhosis, acquired immunodeficiency syndrome, and schizophrenia (Camci et al. 2002; Madsen. et al. 2002; Thom and Petrik 2007,McElvania TeKippe et al. 2012; Canuti et al. 2015; Kazemi et al. 2015; Hettmann et al. 2016; Spandole-Dinu et al. 2018), no significant link was identified with any of these. Furthermore, it has been suggested that anelloviruses positively affect human health by shaping immunity during early development (Kaczorowska and van der Hoek 2020). Thus, anelloviruses are considered to be part of the ‘healthy human virome’ (Arze et al. 2021; Koonin, Dolja, and Krupovic 2021). Apparently, asymptomatic anellovirus infections are also common in other mammals (Fahsbender et al. 2017; Crane et al. 2018; de Souza Wm et al. 2018; Webb, Rakibuzzaman, and Ramamoorthy 2020), suggesting extensive coevolution of anelloviruses with mammalian hosts. Notably, outside of mammals, anelloviruses have been characterized in chickens and several other avian species, and with members of one species, *Gyroviruschickenanemia*, having been shown to cause anemia, intramuscular hemorrhage, lymphoid atrophy, and bone marrow aplasia (Fatoba and Adeleke 2019; Kraberger et al. 2021).

With icosahedral capsids of ∼30 nm in diameter and circular single-stranded (ss) negative-sense DNA genomes of 1.6–3.9 kb (Miyata et al. 1999; Biagini 2009; Biagini et al. 2012), anelloviruses are among the smallest animal DNA viruses. Their genomes include one large open reading frame (ORF1) and several additional short ORFs, typically overlapping with ORF1. The product of ORF1 (∼350–710 amino acids (aa)) has been suggested to function as both the capsid protein (CP) and the replication initiation protein (Rep) (Erker et al. 1999; Mushahwar et al. 1999; Kamahora, Hino, and Miyata 2000; Bendinelli et al. 2001). The N-terminal region of the ORF1 product contains a stretch of positively charged residues, a feature typical of CPs of small RNA and DNA viruses (Rosario, Duffy, and Breitbart 2012; Requião et al. 2020). This protein also has been claimed to contain sequence motifs that are diagnostic of the rolling-circle replication initiation endonucleases of eukaryotic ssDNA viruses (Biagini et al. 2001; Kazlauskas et al. 2019). Recent cryo-electron microscopy analysis has shown that ORF1 of torque teno mini virus (*Betatorquevirus*) contains a jelly-roll domain and indeed forms an icosahedral capsid (Liou et al. 2022). However, the conservation of the jelly-roll domain across the family *Anelloviridae* and the presence or absence of the Rep domain in ORF1 were not investigated. ORF2 (∼100–120 aa), with a conserved N-terminal W-x7-H-x3-C-x-C-x5-H motif (x—any amino acid), has been suggested to function as a phosphatase and a suppressor of the nuclear factor-kappa B pathway (Hijikata, Takahashi, and Mishiro 1999; [Bibr R9][Bibr R9]; Okamoto et al. 2000; Peters et al. 2002; Zheng et al. 2007; Kakkola et al. 2009). Finally, ORF3 (∼70–200 aa) contains a C-terminal serine-rich domain, which has been suggested to be a target for phosphorylation and to play a role in anellovirus infection persistence and possibly in the inhibition of apoptosis (Kamahora, Hino, and Miyata 2000; Asabe et al. 2001; Kooistra et al. 2004; Qiu et al. 2005; Prasetyo et al. 2009; Singh and Ramamoorthy 2016).

None of the anellovirus ORFs bear detectable sequence similarity to proteins of other viruses or any other available proteins. In the absence of an identifiable Rep or a CP typical of other eukaryotic ssDNA viruses, *Anelloviridae* was the only family of eukaryote-infecting viruses with circular ssDNA genomes not included in the phylum *Cressdnaviricota* of the realm *Monodnaviria* (Krupovic et al. 2020). More generally, given the current absence of detected anelloviruses outside of vertebrate hosts and the lack of detectable homology to other known viruses or cellular genes, the evolutionary origins of this group of viruses remain enigmatic.

Based on comparisons of ORF1 protein sequences, known anelloviruses have been classified into 156 species, which are distributed across 30 genera (Biagini 2009; Biagini et al. 2012; Kraberger et al. 2021; Varsani et al. 2021), with 69 percent pairwise nucleotide (nt) identity between ORF1 genes chosen as a species demarcation threshold and genera defined based on the topology of the ORF1 protein phylogenetic tree (Varsani et al. 2021). The most populous genera are *Alphatorquevirus*, *Betatorquevirus*, and *Gammatorquevirus*, which include viruses that infect exclusively primates including humans (Okamoto 2009; Varsani et al. 2021).

Here, we further expand the breadth of known anellovirus diversity by sequencing 256 new complete genomes of anelloviruses from Weddell seals (*Leptonychotes weddellii*) and a grizzly bear (*Ursus arctos horribilis*). A comprehensive family-wide analysis of sequences and the predicted structures of the signature anellovirus protein ORF1 confirmed that they all adopt a jelly-roll fold typical of viral CPs, establishing an evolutionary link to other eukaryotic ssDNA viruses. However, the results of our analysis are inconsistent with ORF1 having any relationship with viral rolling-circle Reps. A comparison of the modeled structures of anellovirus CPs allowed us to reconstruct their evolution through the acquisition and gradual augmentation of a ‘projection domain’ within the ORF1 product, which appears to be at the forefront of virus–host interactions. Collectively, our findings suggest that, whereas the anellovirus ORF1 likely evolved from a canonical single jelly-roll (SJR) CP gene typical of eukaryote-infecting viruses in the phylum *Cressdnaviricota* (Krupovic et al. 2020), the most recent common ancestor of all known anelloviruses apparently lost the Rep-encoding gene.

## Materials and methods

### Sampling, identification, and sequencing of the anellovirus genomes

A single fecal sample from a grizzly bear (*U. arctos horribilis*) was collected in 2016 in Wyoming state (USA). Approximately 5 g of the sample was resuspended in 20 ml of SM buffer (G-Biosciences, USA) (0.1 M NaCl, 50 mM Tris/HCl—pH 7.4, and 10 mM MgSO_4_) buffer and centrifuged at 10,000 *× *g for 10 min. The supernatant was sequentially filtered through 0.45- and 0.2-µm syringe filters, and viral particles in the filtrate were precipitated with 15 per cent w/v PEG 8000 (Sigma, USA). The resulting suspension was incubated overnight at 4°C to precipitate virions and then centrifuged at 6000 *×* g for 20 min. The supernatant was discarded, and the pellet was resuspended in 2 ml of SM buffer. From this suspension, 200 µl was used for viral DNA extraction using the High Pure Viral Nucleic Acid Kit (Roche Diagnostics, USA), and circular DNA molecules in the DNA extract were amplified using rolling-circle amplification (RCA) using the TempliPhi™ kit (GE Healthcare, USA). The RCA product was used to generate a library (TruSeq Nano DNA kit) for paired-end (2 × 100 base pair (bp)) sequencing on an Illumina 4000 sequencer at Macrogen Inc. (Korea).

Nasal and vaginal swabs from adult female Weddell seals (*L. weddellii*) were taken from sedated individuals during the Austral summer seasons of 2015/2016 (*n* = 39) and 2016/2017 (*n* = 40). These samples were collected under National Marine Fisheries Service Marine Mammal permit #17411, Antarctic Conservation Act permit #2014-003, and the University of Alaska Anchorage and the University of Alaska Fairbank’s Institutional Animal Care and Use Committee approvals #419971 and #854089. Following sampling, the vaginal and nasal swabs were stored at 4°C in UTM™ Viral Transport Media (Copan, USA). An aliquot of 1 ml of the transport media was filtered through a 0.2-µm syringe filter, and 200 µl of the filtrate was used for viral DNA extraction using the High Pure Viral Nucleic Acid Kit (Roche Diagnostics). Circular DNA molecules in the extract were amplified using RCA using the TempliPhi™ kit (GE Healthcare). For each of the two seasons, a 5 µl aliquot of the RCA product was pooled for nasal and vaginal swabs and used to generate four libraries (TruSeq Nano DNA kit) for paired-end (2 × 100 bp) sequencing on an Illumina 4000 sequencer at Macrogen Inc. (Korea).

The 2 × 100 bp raw reads from the five libraries were trimmed with Trimmomatic v0.39 (Bolger, Lohse, and Usadel 2014) and then *de novo* assembled using metaSPades v3.14 (Bankevich et al. 2012). The *de novo* assembled contigs (>1,000 nts) were checked for terminal redundancy to determine if they represent circular molecules. All contigs >1,000 nts were analyzed using BLASTx (Altschul et al., 1990) against a RefSeq viral protein database (RefSeq release 205). For all contigs that we identified as anellovirus-like, we assembled a dataset and designed sets of abutting primers (Supplementary Table S1) to recover complete genomes from each of the individual samples of the Weddell seal nasal and vaginal swabs and the grizzly bear fecal sample. The RCA products were used as templates with specific primer pairs to amplify the anellovirus genomes using Kapa HiFi Hotstart DNA polymerase with the following thermal cycling conditions: 95°C for 3 min; 25 cycles of 98°C for 20 s, 60°C for 15 s, 72°C for 2 min, and a final extension of 72°C for 3 min. The amplicons were resolved on a 0.7 per cent agarose gel stained with SYBR Safe (ThermoFisher, USA), and ∼2–2.5 kb size fragments were excised, gel purified, and cloned into pJET1.2 plasmid vector (ThermoFisher) and transformed into *Escherichia coli* XL1-blue competent cells. The resulting recombinant plasmids were Sanger sequenced by primer walking at Macrogen Inc. (South Korea).

ORFs in the cloned and Sanger sequenced genomes were identified using ORF finder tool in Geneious Prime 2022.1.1 (Biomatters Ltd, New Zealand) and manually checked by global alignments. The sequences have been deposited in GenBank under accession numbers OP629190–OP629445.

Pairwise identity analysis to determine species classification was performed using SDT v 1.2 (Muhire, Varsani, and Martin 2014).

### Recombination analyses

The 246 sequences of the anelloviruses identified from Weddell seal samples in this study represent two anellovirus species *Torque teno pinniped virus 8* (*n* = 160) and *Torque teno pinniped virus 9* (*n* = 86), and thus, these were aligned together with other members of these two species using Multiple Alignment using Fast Fourier Transform (MAFFT) with auto option (Katoh and Standley 2013). Recombination in the aligned datasets was inferred using Recombination Detection Program 5 (RDP5) (Martin et al. 2021) with default settings. Sequences were auto-masked for optimal recombination detection, and only events detected with more than three different methods implemented in RDP5 coupled with phylogenetic support for recombination and a *P*-value of <0.05 were considered credible.

The ten anelloviruses derived from the grizzly bear feces are diverse (each representing a new species sharing <69 per cent ORF1 identity) and thus could not be reliably aligned and analyzed for recombination with other closely related anelloviruses.

### Dataset of anellovirus proteins

Sequences representing all anellovirus species (*n* = 153) listed in the Virus Metadata Resource provided by the International Committee on Taxonomy of Viruses (ICTV) were downloaded from the National Center for Biotechnology Information. This reference dataset was supplemented with the newly sequenced anelloviruses from Weddell seals (*n* = 246) and grizzly bears (*n* = 10) (Supplementary Tables S2 and 3). Three main ORFs, ORF1-3, and hypothetical ORFs from Weddell seal anelloviruses (WSAs) were extracted from the annotated sequence files in Geneious Prime 2022.1.1 (Biomatters Ltd) and used in the subsequent analyses.

### Phylogenetic analysis

For taxonomic and phylogenetic analyses, the ORF1 protein sequences of representative anelloviruses (one per species) together with the 256 from this study were clustered to 80 per cent identity with CD-HIT (Li and Godzik 2006) and aligned using MAFFT with G-INS-i option (Katoh and Standley 2013). The alignment was trimmed with TrimAL (Capella-Gutierrez, Silla-Martinez, and Gabaldon 2009) with gap = 0.2 option, and the resulting alignment was used to infer a maximum-likelihood phylogenetic tree with PhyML 3.0 (Guindon et al. 2010) with VT+G+ F as the amino acid substitution model determined using ProtTest (Darriba et al. 2011) and SH-like branch support. The tree was annotated and visualized in iTOL (Letunic and Bork 2021).

The sizes of anellovirus genomes and projection domains were mapped onto a tree of their respective host species obtained from TimeTree of Life (Kumar et al. 2022).

### Structural modeling and similarity search

For representatives of each anellovirus genus, including the new WSA and grizzly bear anellovirus (GBA), protein sequences of ORFs 1, 2, or 3 were used as inputs for AlphaFold2 (version 2.1.1; Jumper et al. 2021) structural prediction. The quality of generated structural models was assessed using the local distance difference test (Mariani et al. 2013) (Supplementary Fig. 1). All ORF1 models generated in this study are provided in Supplementary Data File 1.

The predicted structures of ORFs 1, 2, and 3 were used as inputs for the DALI (Holm 2020) server, and the structural similarity was evaluated based on the DALI Z score, where scores above 2 are considered potentially significant (Holm 2020). For ORF1 homologs with large spike insertions, the jelly-roll and spike domains were split, and DALI searches were run separately. Only the top 20 hits against the Protein Data Bank (PDB50) database were extracted. To build the structure-based dendrogram, structures of ORF1 and their top DALI hits were used in an all-against-all comparison, and an average linkage clustering was performed via DALI.

The protein sequences representing each anellovirus genus were used as queries in profile–profile comparisons with HHsearch (Steinegger et al. 2019) against the PDB70 and viral protein (UniProt-SwissProt-viral70_3_Nov_2021) profile databases (Gabler et al. 2020).

### Search for Rep protein motifs

To search for the potential anellovirus Rep homologs, all six possible frames were *in silico* translated from representative anellovirus genomes using Prodigal (Hyatt et al. 2010) in meta mode and five different translation tables: standard code; vertebrate mitochondrial code; yeast mitochondrial code; invertebrate mitochondrial code, and bacterial, archaeal, and plant plastid code. These resulting amino acid sequences were used as input for InterProScan (Jones et al. 2014) against all available databases with default settings.

## Results and discussion

### New anelloviruses from Weddell seals and grizzly bears

**Figure 1.**
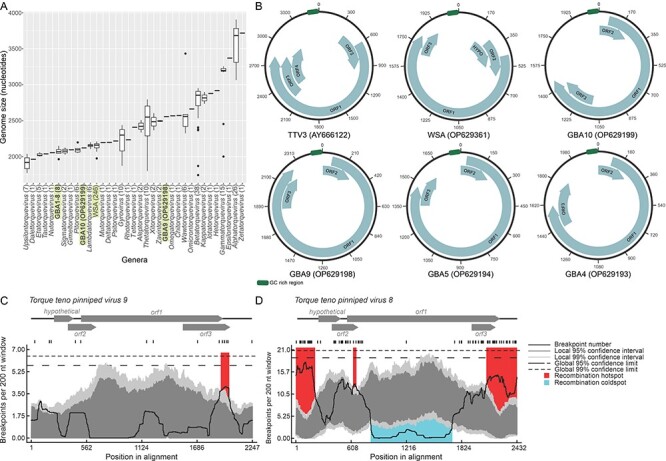
Genomic characteristics of new anelloviruses. (A) A barplot of genome sizes per anellovirus genera. The number of genomes in each category is provided in parentheses. (B) Genome maps of a prototypical anellovirus, TTV3, as well as a representative WSA and four grizzly bear anelloviruses. (C–D) The recombination analysis within the anellovirus species *Torque teno pinniped virus 9* (C) and *Torque teno pinniped virus 8* (D), with breakpoint hotspots shown in red and cold spots in blue. The dark gray and light gray areas of the plots indicate 95 and 99 per cent confidence intervals, respectively. Detectable breakpoint positions are shown with vertical lines above the graphs. The thick black line is the plot of the number of breakpoints detected within the 200-nt window region, and the window was moved along each of the represented alignments 1 nt at a time.

The newly sequenced genomes of WSAs and GBAs range from ∼2,000 to 2,600 nts in length and are on the smaller side of the anellovirus genome size distribution (Fig. 1A). The WSA genomes display uniform sizes similar to those of viruses in the genus *Lambdatorquevirus* (∼2,150 nts; Fig. 1A), with the exception of WSA isolate N1637_a (OP629327), which has a considerably shorter genome of 1,976 nts, with a 210-nt region in the ORF1 (5′ region) missing. The latter sequence might therefore be a subgenomic molecule. By contrast, the GBA genomes are more variable, ranging from 1,965 to 2,557 nts (Fig. 1A, B). The WSA and GBA genomes display an organization similar to that of other anelloviruses, with one large (∼1,200 nts) and several shorter (∼200 nts) ORFs, three ORFs in the WSA genome sequences, and two in the GBA genome sequences (Fig. 1B). Similar to other anelloviruses, the WSA and GBA genomes contain a GC-rich sequence in the intergenic region (Fig. 1B) that likely plays a role in genome replication.

**Figure 2.**
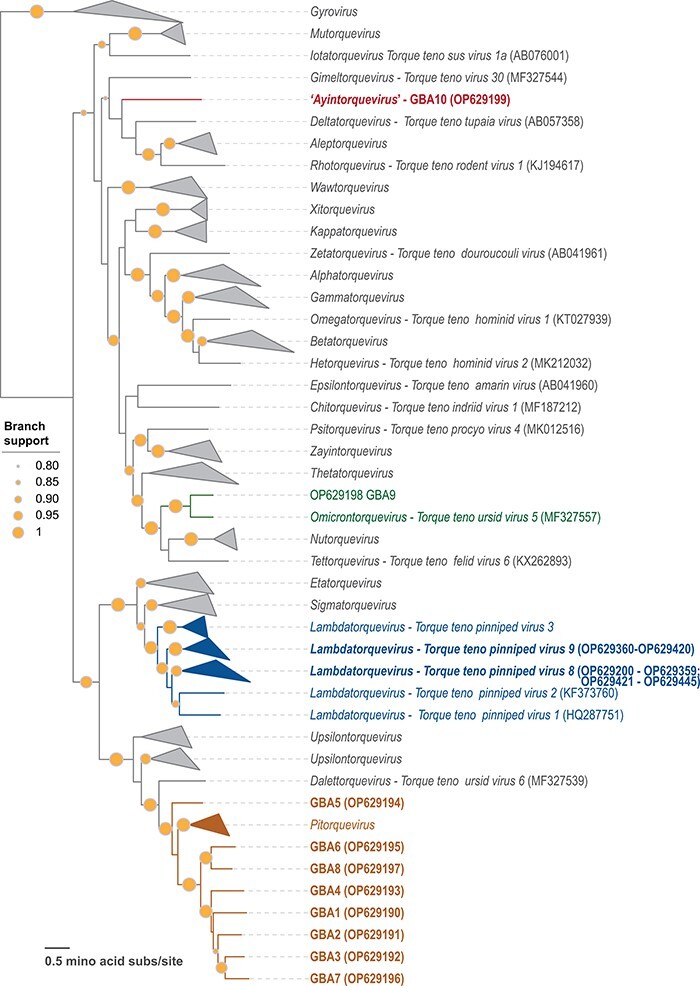
The maximum-likelihood phylogenetic tree of ORF1 protein sequences of anelloviruses representing each of the established anellovirus species and those from the newly sequenced Weddell seal and grizzly bear anelloviruses. Branches representing species of particular genera to which the ORF1s of the anelloviruses identified in this study are not part of have been collapsed. In cases where there is only one species in a genus, the genus and the species names (with exemplar sequence accession number) are provided. The bold taxa names represent the ORF1s of the anelloviruses from this study, and the genera they belong to have been color coded. Branches with >0.8 SH-like support are shown.

Maximum-likelihood phylogenetic analysis based on the ORF1 amino acid sequences from the anelloviruses representing each of the established species from the 31 genera and the new anelloviruses sequenced in this study (*n* = 409; Supplementary Files 1 and 2) largely recapitulated the established ICTV taxonomy (Fig. 2). Based on ORF1 nt pairwise identities and phylogenetic analysis, the WSA sequences belong to two species *Torque teno pinniped virus 8* (*n* = 160) and *Torque teno pinniped virus 9* (*n* = 86) in the genus *Lambdatorquevirus*. Both these species include anelloviruses that have been previously sampled only from Weddell seals (Fahsbender et al. 2017). By contrast, the GBA sequences belong to the *Pitorquevirus* (*n* = 8) and *Omicrontorquevirus* (*n* = 1) genera, and one is likely a member of a new genus. In particular, seven genomes, GBA-1, -2, -3, -4, -6, -7, and -8 (OP629190, OP629191, OP629192, OP629193, OP629195, OP629196, and OP629197), form a clade that is a sister group to other classified pitorqueviruses, all of which were sampled from giant pandas (*Ailuropoda melanoleuca*) (Zhang et al. 2017). The GBA-10 sequence (OP629199) branches separately from viruses in the established genera and hence can be considered a founding member of a new genus, which we propose to name ‘*Ayintorquevirus*’ (Fig. 2). Based on the pairwise identities of the ORF1s of the GBAs, which share <69 per cent identity with those of other anelloviruses, each of these represents a new species.

In viruses of the species *Torque teno pinniped virus 9*, there is a recombination hotspot in the 3′ region of the *orf1* and *orf3* genes, which is similar to that reported previously (Fahsbender et al. 2017) (Fig. 1C). Notably, an equivalent hotspot is also present in viruses of the species *Torque teno pinniped virus 8*, where it not only encompasses the 3′ of the *orf1* and *orf3* genes but extends to the entire non-coding intergenic region. An additional small hotspot was detected at the 3ʹ end of *orf2* and 5ʹend of *orf1* genes (Fig. 1D, Supplementary Table 4). Conversely, the middle of the *orf1* gene, spanning ∼800–1,700 nts of the genome alignment, is a recombination cold spot (Fig. 1D). These observations strongly suggest that the termini of *orf1* as well as the non-coding region can be exchanged through recombination between anellovirus genomes, whereas the middle region is protected from such exchanges, possibly, due to strong purifying selection on the protein structure.

### Anellovirus ORF1 is a homolog of SJR CPs

To analyze the relationship between anelloviruses and other viruses, we performed BLASTp searches against the viral RefSeq database queried with ORF1 protein sequences representing each of the *Anelloviridae* genera. No significant matches were obtained outside of the *Anelloviridae*. Thus, we sought to gain insights into the provenance of anelloviruses through more sensitive profile–profile comparisons. Searches queried with the ORF1 sequence of torque teno virus 1 (TTV1; NP_817122) against the PDB and viral protein profile databases using HHsearch yielded a highly significant hit (probability = 95.8) to the CP of a bat circovirus (PDB id: 6RPO; Supplementary Table S5). Importantly, the aligned regions encompassed nearly the entirety of the circovirus CP but only ∼30 per cent of the anellovirus protein, highlighting the disparity in their respective sizes (233 versus 770 aa). Significant hits were also obtained with ORF1 sequences from viruses of other anellovirus genera (Supplementary Table S5). Notably, however, not all anellovirus ORF1 sequences yielded global alignments. For instance, a search queried with the more compact ORF1 of chicken anemia virus (449 aa) retrieved only a match to the N-terminal half of the circovirus CP, indicating a higher divergence between these proteins.

**Figure 3.**
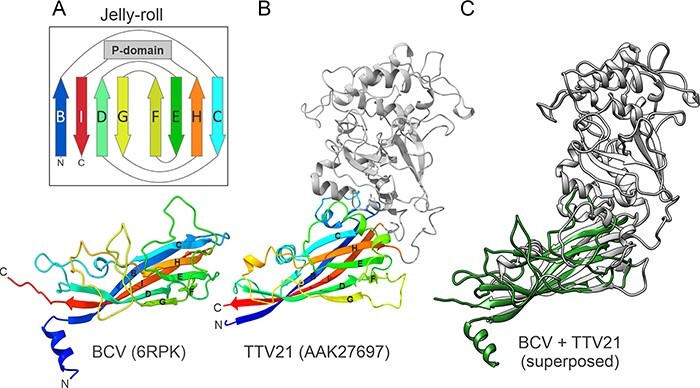
The structure of the circovirus jelly-roll CP and structural model of anellovirus ORF1. (A) CP of bat circovirus (BCV, family *Circoviridae*; PDB id: 6RPK) colored using the rainbow scheme from blue N-terminus to red C-terminus. The insert shows the schematic topology of the jelly-roll fold. The eight β-strands are labeled from B to I and form two antiparallel β-sheets BIDG and CHEF. The location of the projection (P)-domain is shown as a gray rectangle. (B) The structural model of ORF1 from TTV 21 (genus *Alphatorquevirus*; GenBank accession number: AAK27697). The jelly-roll domain shared with circoviruses is colored using the rainbow scheme, whereas the P-domain is shown in gray. (C) The superposed structures of the BCV (green) and TTV21 (gray) structures.

Circovirus CPs contain an N-terminal arginine-rich region involved in DNA binding (Erker et al. 1999; Sarker et al. 2016) and a jelly-roll domain comprising eight antiparallel β-strands (B through I), which form two juxtaposed β-sheets, BIDG and CHEF (Fig. 3A) (Sarker et al. 2016; Khayat et al. 2019). Structurally similar CPs with the SJR fold are encoded by a vast diversity of eukaryote-infecting ssDNA and ssRNA viruses with icosahedral capsids (Rossmann and Johnson 1989; Krupovic and Koonin 2017). Notably, circovirus CPs have one of the most compact SJR cores, with no structural elaborations, such as long loops connecting the β-strands, additional secondary structure elements, or even distinct domains that are occasionally found to be inserted into CPs of other viruses (Wery et al. 1994; Wada et al. 2008; Kazlauskas et al. 2017; Munke et al. 2022).

The anellovirus ORF1 protein also contains an arginine-rich N-terminal region similar to that of circovirus CPs. To verify to what extent the structural similarity extends to the SJR domain, representative ORF1 protein sequences from each of the established *Anelloviridae* genera as well as those of the newly sequenced WSA and GBA genomes were modeled with AlphaFold2 (Jumper et al. 2021). Inspection of the generated models confirmed that all ORF1 protein homologs contained the conserved SJR domain (Fig. 3B, C, Supplementary Fig. 2). Indeed, DALI (Holm 2020) searches against the PDB database queried with the predicted jelly-roll domains of different ORF1 protein orthologs consistently yielded best hits, with significant *Z*-scores (Z = 6.0–9.6), to the CPs of eukaryotic ssDNA and ssRNA viruses from the families *Circoviridae*, *Hepeviridae*, *Astroviridae*, *Marnaviridae*, *Nodaviridae*, and *Dicistroviridae*, and the genus *Papanivirus* (Supplementary Table S6). Collectively, the results of profile–profile and structure comparisons indicate that the anellovirus ORF1 protein is homologous to the jelly-roll CPs of other eukaryote-infecting ssDNA viruses and, in particular, to the CPs of circoviruses.

We also modeled the structures of the anellovirus ORF2 and ORF3 as well as hypothetical proteins. However, in all cases, these short proteins yielded structures with generic, simple folds, such as helix-turn-helix, which gave no useful information on their provenance (Supplementary Fig. S3).

### Structural dissection of the anellovirus ORF1 proteins

Anellovirus ORF1 proteins display a remarkable variation in length and sequence. To verify whether these proteins are orthologous, we performed an all-against-all structural comparison of the ORF1 homologs from different *Anelloviridae* genera and CPs of other viruses identified as the top hits in the DALI searches.

**Figure 4.**
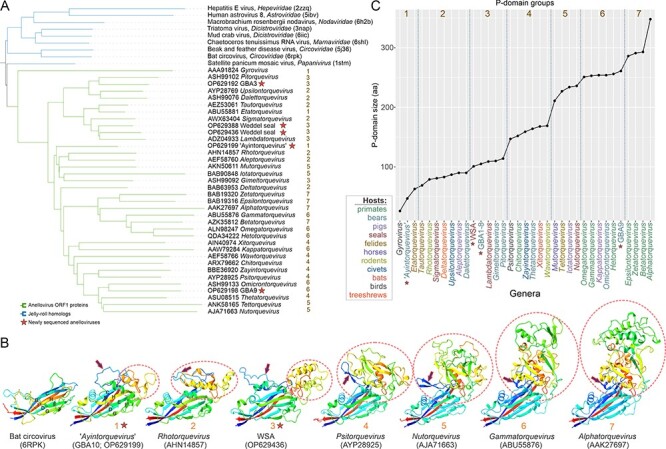
The evolution of the anellovirus CP by the projection domain augmentation. (A) The average linkage dendrogram of anellovirus ORF1 structures and SJR CP homologs obtained from the DALI server. The homologous CP branches are marked in blue, and the anellovirus ORF1 structures are marked in green. (B) Examples of ORF1 structures with the circled projection domains and an arrow indicating the structure between strands B and C are also shown. The stars mark each structure from the newly sequenced anelloviruses in the dendrogram. The models are colored using the rainbow scheme, from blue N-terminus to red C-terminus. (C) The plot of spike domain sizes from each anellovirus genus. The colors mark the host of the viruses.

**Figure 5.**
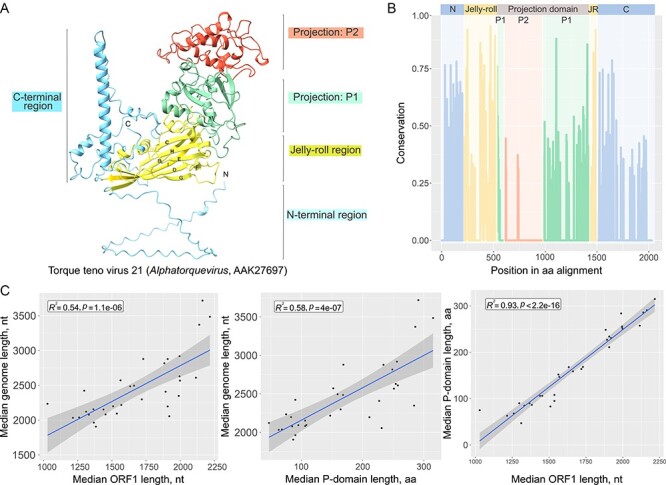
The conservation of the jelly-roll and extensive divergence of the projection domain in the anellovirus CPs. (A) The structural model of TTV 21 ORF1 (AAK27697). Different regions of ORF1 are highlighted using different colors. (B) Shannon conservation index plotted for each amino acid position in the ORF1 alignment. High lines mark high conservation and low lines mark low conservation. Domains of the ORF1 structure as in A are marked. (c) Correlation plots of genome sizes, ORF1 sizes, and P-domain sizes. The black dots correspond to median values per genus.

This analysis showed that all anelloviruses form a compact, arguably, monophyletic group (Fig. 4A), suggesting that the dramatic evolution observed among the ORF1 sequences took place following their divergence from a common ancestor. To reconstruct the gradual evolution of the ORF1 structure, we superposed the structures of ORF1 homologs from different genera with the compact CP of a circovirus (PDB id: 6RPK; Fig. 4B). This comparison allowed further delineating the borders of the anellovirus jelly-roll domain and exposed the differences compared to the SJR CP of circoviruses. In particular, most ORF1 proteins contain two prominent insertions. One is located between β-strands B and C (Fig. 4B) and, depending on the virus, can adopt an extended loop, α-helix or β-hairpin conformation. The other insertion is located within a loop connecting the β-strands H and I. The H-I insertion varies substantially in both size and predicted structure, from an extended loop to an elaborate α + β domain (Fig. 4B, C). Structural searches queried with the latter domain did not produce significant matches against the PDB database, suggesting that this fold is so far unique to anelloviruses. Notably, the relative orientation of the α + β domain with respect to the jelly-roll domain is similar to the ‘projection’ (P) domains present in the CPs of a variety of ssDNA and ssRNA viruses, such as bacilladnaviruses, tombusviruses, nodaviruses, caliciviruses, and several other groups of viruses (Wery et al. 1994; Wada et al. 2008; Kazlauskas et al. 2017; Munke et al. 2022). In all these viruses, the projection domains face away from the capsid surface and, at least in some cases, are implicated in host recognition and binding (Tan, Hegde, and Jiang 2004). This prediction is confirmed by the recent cryogenic electron microscopy structure of the betatorquevirus LY1 (Liou et al. 2022). It is plausible, therefore, that the P-domains of anellovirus ORF1 proteins play a similar role, despite the absence of sequence or structural similarity to the equivalent domains found in the CPs of other viruses. Consistent with the role of the P-domain in host recognition/interaction, mapping of the sequence conservation (Shannon diversity) on the ORF1 structure revealed a high variability in the outermost region of the P-domain. Thus, based on sequence divergence, the P-domain can be subdivided into the SJR-proximal P1 and the SJR-distal, highly divergent P2 subdomains. The latter region has been previously noted to be hypervariable in anelloviruses (Nishizawa et al. 1999; Jelcic et al. 2004; Arze et al. 2021; Kaczorowska et al. 2023), and our findings provide a biological rationale for its high evolution rate. Notably, the same region was identified in our analysis as a recombinational cold spot in *Torque teno pinniped virus 8* (Fig. 1D), suggesting that the P-domain primarily evolves through substitutions rather than recombination, which is likely to be suppressed by natural selection, conceivably, due to the selection for the preservation of structurally important amino acid interactions in this domain. Unlike the P-domain, and especially, the P2 subdomain, which appears to co-evolve with the host factors following Red Queen dynamics, the evolution of the SJR domain is constrained by its likely role in capsid shell formation and is accordingly considerably slower (Fig. 5A, B). A similar distribution of conservation across the SJR and P-domains has been also observed for other ssDNA viruses, e.g. ‘cruciviruses’ (Roux et al. 2013; Quaiser et al. 2016; de la Higuera I et al. 2020).

### Evolution of ORF1 through gradual augmentation of the P-domain

There is a notable correlation between the anellovirus genome size, ORF1 length, and the size of the P-domain (*R*^2^ = 0.92 for ORF1-P-domain size and >0.5 for the rest of the combinations; *P*-value <2.2e-16 for ORF1-P-domain size and <1e-06 for the rest of the combinations; and Spearman rank correlation coefficient = 0.97 for ORF1-P-domain size and >0.72 for the rest of the combinations). These correlations indicate that the increase in the genome size is almost exclusively attributable to elaborations within the P-domain (Fig. 5C). Based on the size of the P-domain, anelloviruses can be classified into seven groups (Fig. 4C). Group 1 includes anelloviruses with the most compact ORF1 proteins, among which members of the genus *Gyrovirus*, the only group of anelloviruses known to infect birds, have the smallest ORF1. The ORF1 proteins of gyroviruses, such as chicken anemia virus, lack the P-domain, and instead, the β-strands of the SJR core are connected through extended loops, which occasionally contain short α-helices. In the structure-based tree, gyrovirus ORF1 forms a sister group to mammalian anelloviruses (Fig. 4A), suggesting an early divergence. Among the mammalian anelloviruses, one of the newly sequenced GBA, GBA-10 (OP629199), and members of the genus *Etatorquevirus*, which are most similar to members of the genus *Gyrovirus* in *Anelloviridae* family, also lack an elaborate P-domain, and instead, β-strands H and I are connected through an extended loop with a short α-helix. Extensive loops connecting the β-strands of the SJR domain are also typical of viruses with linear ssDNA genome in the family *Parvoviridae*, such as adeno-associated virus and canine parvovirus (de Villiers and Hausen 2009; Grekova et al. 2012). However, unlike in anelloviruses, the extended loops in parvovirus CPs are located between β-strands G and H.

The predicted ORF1 structures in the six other anellovirus groups display a gradual complexification of the P-domain through (1) increase in the α-helical content within the H-I loop in Groups 2 and 3, which can be considered as the emergence of the P1 subdomain; (2) appearance of a four-stranded β-sheet in Group 4; (3) acquisition of additional β-sheets in Groups 5 and 6; (4) further proliferation of the unstructured loops and secondary structure elements in the SJR-distal region of the P1 subdomain in Group 6, signifying the emergence of the P2 subdomain; and (5) in Group 7, the P2 subdomain reaches the peak of its complexity, becoming more structured, with an increased α-helical content. The existence of such a broad range of structural complexity in ORF1 proteins of contemporary anelloviruses appears surprising, and its biological implications, in particular, for virus–host interactions, remain to be explored. Furthermore, it would be interesting to determine whether the increasingly augmented P-domains represent intermediate evolutionary steps, or these elaborations evolved independently in different lineages.

Seven of the ten anellovirus genera with the largest P-domains (ORF1 Groups 6 and 7) include viruses infecting primates (*Alpha-, Beta-, Gamma-, Epsilon-, Zeta-, Omega*-, and *Hetorquevirus* genera), whereas the remaining three genera, all in Group 6, are associated with bears and pigs (*Omicron*- and *Kappatorquevirus*, respectively) (Fig. 4C). Viruses from the remaining 20 *Anelloviridae* genera showed no recognizable patterns of clustering based on the P-domain size and the host. To further explore this relationship, we mapped the anellovirus genome and P-domain sizes onto a phylogenetic tree of their respective hosts derived from the TimeTree of Life website (http://www.timetree.org/). This analysis further highlighted that primate-infecting anelloviruses have considerably larger genomes and P-domains compared to any other group of hosts (Supplementary Fig. S4). A distinguishing feature of ORF1 proteins in Groups 6 and 7 is the presence of the P2 subdomain, suggesting that it might play an important role in the evasion of the primate immune response and/or the recognition of primate-specific host receptors. What differences between the immune systems of primates and those of other mammals might be driving this difference remains unclear.

### Outstanding questions about the anellovirus genome replication

As mentioned earlier, it has been previously reported that the ORF1 protein of betatorqueviruses contains conserved motifs indicative of it potentially being related to the rolling-circle replication initiation endonucleases of the HUH superfamily (Biagini et al. 2001; Umemura et al. 2002; Huang et al. 2010). However, these motifs were subsequently not found to be conserved throughout the family (Rosario, Duffy, and Breitbart 2012). The structural model of ORF1 from torque teno mini virus 1 (*Betatorquevirus*) allows us to reevaluate the relevance and validity of the ‘Rep motifs’ in the structural context. The HUH superfamily Rep proteins have the RNA-recognition-motif (RRM) fold, similar to the Palm domain of RNA and DNA polymerases (Umemura et al. 2002), with the conserved Motifs I and II being located within Strands β1 and β3, respectively, and the invariable catalytic tyrosine residue of Motif III located within an α-helix (Krupovic, Dolja, and Koonin 2019; Tarasova and Khayat 2021). It is obvious from our structural model that no part of the anellovirus ORF1 adopts the RRM fold. Furthermore, the putative Rep motifs are located within the P-domain, with ‘Motifs I and II’ mapping to spatially separated loops and ‘Motif III’ residing within a β-strand (Supplementary Fig. S5). Thus, beyond any reasonable doubt, the ‘RCR motifs’ in ORF1 do not signify ORF1 homology to HUH Reps of other ssDNA viruses and therefore cannot play equivalent roles to those of the HUH Reps during viral genome replication. Other proteins encoded by anelloviruses, even when alternative coding tables are used for *in silico* protein translation, did not show any similarity to known replication proteins either.

Thus, the question of how anelloviruses replicate their genomes remains open and enigmatic. In particular, it is unclear which viral and/or cellular factors are involved in the initiation of anellovirus replication. One possibility is that, similar to satellite viruses (Krupovic, Kuhn, and Fischer 2016), anelloviruses depend for replication on a coinfecting virus, which would provide the Rep protein in trans. Indeed, it has been shown that TTV-HD14 genome replication was enhanced during coinfection with Epstein–Barr virus (*Othoherpesviridae*) or in a cell line expressing the large T-antigen of simian virus 40 (*Polyomaviridae*) (de Villiers Em et al. 2011; Borkosky et al. 2012). However, neither orthoherpesviruses nor polyomaviruses produce ss replication intermediates, suggesting that the positive effect of these viruses on anellovirus replication was indirect. Furthermore, the importance of a helper virus appears unlikely, given the recent breakthrough in achieving anellovirus replication and virion formation *in vitro* in the T cell–derived human cell line MOLT-4 (Nawandar et al. 2022). Transfection of the MOLT-4 cells with a plasmid comprising a tandem anellovirus genome yielded unit-length, replicated anellovirus genomes, which were packaged into isometric virus particles. This result indicates that all factors required for genome replication, its conversion into an ssDNA form, and packaging are provided by the virus and/or the host. Alternatively, the Rep function could be provided in trans by the rolling-circle transposons, such as Helitrons (Kapitonov and Jurka 2001) or their fish relatives Heletrons (Poulter, Goodwin, and Butler 2003). However, although functional helitrons are found in bats (Kosek et al. 2021), no active copies have been described in any primates. Previous studies have documented the accumulation of subgenomic anellovirus DNA molecules smaller than the size of the wild-type virus genome, which could be by-products of recombination-dependent replication (Leppik et al. 2007; de Villiers Em et al. 2011), a replication mechanism alternative to RCR that has been described in circular ssDNA viruses in the family *Geminiviridae* (Jeske 2001; Preiss and Jeske 2003; Alberter, Ali Rezaian, and Jeske 2005; Jovel, Preiß, and Jeske 2007; Erdmann et al. 2010). In this mode of replication, the host DNA polymerase produces a covalently closed double-stranded circular form of the viral genome and then generates long concatemers using this dsDNA as a template. The linear genome concatemers produced from the template are then resolved as double-stranded circular molecules by homologous recombination. Thus, conceivably, anelloviruses could rely exclusively on recombination-dependent replication. However, how the dsDNA intermediates would be converted into the ssDNA mature genomes that are eventually packaged is unclear. The availability of the transfection system for anelloviruses will be instrumental in elucidating the mechanisms of anellovirus genome replication.

## Concluding remarks

Here, we explored the evolution of anelloviruses through a comparative structural analysis of their ORF1 proteins. We showed that ORF1, the signature protein of anelloviruses, has the jelly-roll fold, the most common fold in the CPs of viruses with small icosahedral capsids. While this manuscript was in preparation, a preprint describing the structure of ORF1 from the human betatorquevirus LY1 was posted online (Liou et al. 2022). Although the structural model was not publicly available at the time of this writing, precluding direct comparison, the experimentally determined structure appears to be closely similar to the betatorquevirus ORF1 models generated herein using AlphaFold2. There is extensive variability in the overall size of ORF1 protein of anelloviruses belonging to different genera, with the jelly-roll domain being highly conserved, consistent with its key role in virus particle formation, whereas the P-domain is highly variable. Systematic comparison of ORF1 protein structures across the family *Anelloviridae* suggests that anellovirus ORF1 protein evolved from an ancestral virus with a more compact CP, through incremental augmentation and complexification of the ORF1 protein structure. Sensitive profile–profile comparisons have shown that anellovirus ORF1 proteins are homologous to the CPs of circoviruses, currently the only other known group of viruses with small circular ssDNA genomes that infect mammals (Krupovic et al. 2020, 2020). Given the compact structure of the circovirus CPs, the canonical circovirus genome organization, with the *cp* and *rep* genes, similar to that of other ssDNA viruses, it appears likely that anelloviruses evolved in the vertebrate hosts from a circovirus-like ancestor. However, whether this virus was a member of the extant family *Circoviridae* or was ancestral to both circoviruses and anelloviruses remains unclear due to the high sequence divergence. Under this scenario, the emergence of the anellovirus ancestor would entail a dramatic change in the genome replication strategy, accompanied by the loss of the *rep* gene, a feature that sets anelloviruses apart from all other viruses in the phylum *Cressdnaviricota* (Krupovic et al. 2020). Notably, bidnaviruses have apparently evolved along a similar evolutionary path from parvoviruses (both classified in the phylum *Cossaviricota*) by replacing the HUH superfamily Rep with a protein-primed family B DNA polymerase (Krupovic and Koonin 2014). An alternative scenario that could be consistent with the available data involves the transfer of the capsid gene from a circovirus-like ancestor to a distinct replicon that replicated without the involvement of an HUH-Rep. However, such non-viral replicons related to anelloviruses are not known, so that, in the absence of understanding of the anellovirus genome replication mechanism as well as cellular or viral replication determinants, this scenario cannot be currently substantiated.

Due to the lack of identifiable relationship to other known ssDNA viruses, anelloviruses so far have not been considered as belonging to the realm *Monodnaviria* (Koonin et al. 2020). However, in light of our current results, anelloviruses arguably should be included in this realm, probably, as a separate new phylum within the kingdom *Shotokuvirae*, alongside *Cressdnaviricota* and *Cossaviricota*. Thus, we propose assigning *Anelloviridae* to the order ‘*Sanitavirales*’ (after Latin *sanitas* for health, referring to anelloviruses being part of the healthy human virome), within the class ‘*Cardeaviricetes*’ (after Cardea, the Roman goddess of health) and phylum *Commensaviricota*, referring to the commensal lifestyle of anelloviruses.

## Supplementary Material

vead035_Supp
