# Supplementary material for: Evolution of anelloviruses from a circovirus-like ancestor through gradual augmentation of the jelly-roll capsid protein
Source: Virus Evol. 2023 May 27;9(1):vead035. doi: 10.1093/ve/vead035 (PMC10266747; doi:10.1093/ve/vead035)
Supplement: vead035_Supp [file vead035_supp.zip › suppl_data/SUPPLEMENTARY INFORMATION.pdf]

SUPPLEMENTARY INFORMATION

Evolution of anelloviruses from a circovirus-like ancestor through gradual augmentation of the jelly-roll capsid protein

Anamarija Butkovic[1], Simona Kraberger[2], Zoe Smeele[2], Darren P Martin[3], Kara Schmidlin[2], Rafaela S Fontenele[2], Michelle R Shero[4], Roxanne S Beltran[5], Amy L Kirkham[6], Maketalena Aleamotu'a[7], Jennifer M Burns[8], Eugene V. Koonin[9], Arvind Varsani[2]*, Mart Krupovic[1]*

[1] Institut Pasteur, Université Paris Cité, CNRS UMR6047, Archaeal Virology Unit, 25 rue du Dr Roux, 75015 Paris,
[2] The Biodesign Center for Fundamental and Applied Microbiomics, Center for Evolution and Medicine, School of Life Sciences, Arizona State University, Tempe, AZ 85287, USA
[3] Computational Biology Division, Department of Integrative Biomedical Sciences, Institute of Infectious Diseases and Molecular Medicine, University of Cape Town, Observatory 7925, South Africa
[4] Biology Department, Woods Hole Oceanographic Institution, 266 Woods Hole Rd, Woods Hole, MA 02543, USA
[5] Department of Ecology and Evolutionary Biology, University of California Santa Cruz 130 McAllister Way, Santa Cruz CA 95060, USA
[6] U.S. Fish and Wildlife Service, Marine Mammals Management, 1011 E. Tudor Road, Anchorage, AK 99503, USA
[7] School of Environmental and Life Sciences, The University of Newcastle, Callaghan, NSW 2308, Australia
[8] Department of Biological Sciences, Texas Tech University, Lubbock, TX 79409, USA
[9] National Center for Biotechnology Information, National Library of Medicine, Bethesda, MD, USA

For correspondence:
Arvind Varsani, E-mail: arvind.varsani@asu.edu
Mart Krupovic, E-mail: mart.krupovic@pasteur.fr

Supplementary figures

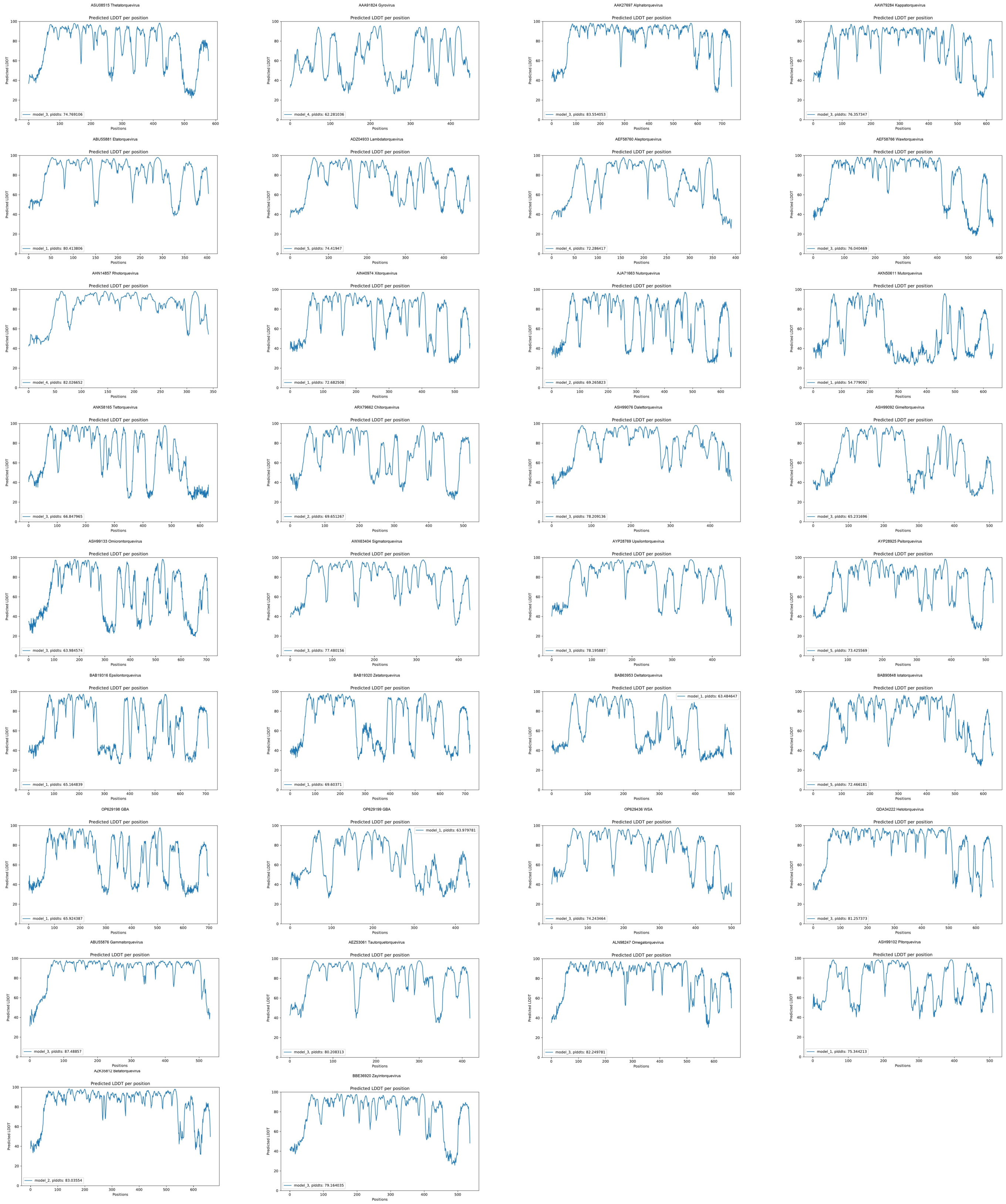

Supplementary Figure S1. The per-residue confidence scores for AlphaFold2 structural models of ORF1 estimated using the local distance difference test (lDDT). Regions with lDDT > 90 are expected to be modelled to high accuracy, whereas regions with lDDT between 70 and 90 are expected to be modelled well (a generally good backbone prediction).

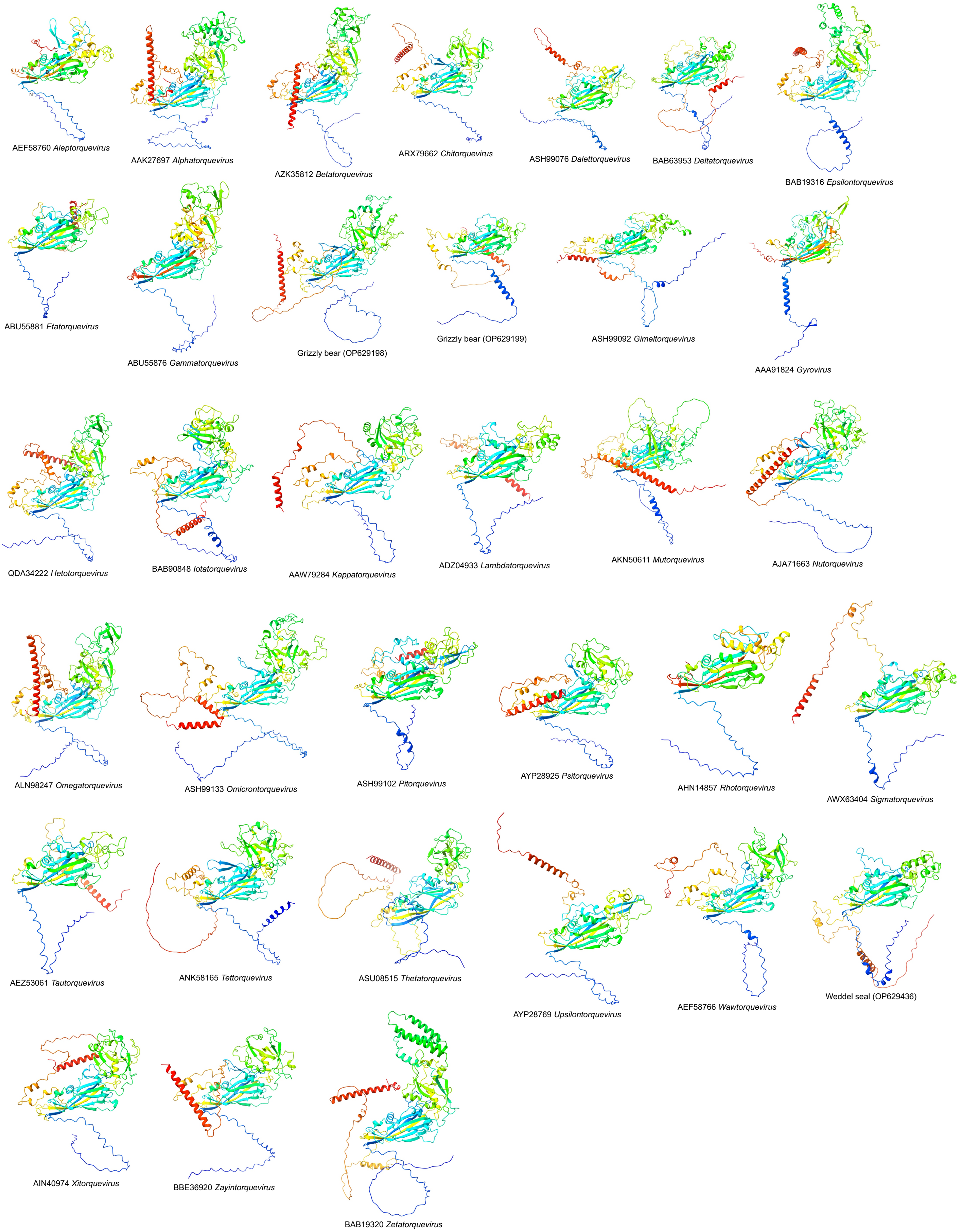

Supplementary Figure S2. The modelled anellovirus structures, one per genus, sorted alphabetically. The models are colored using the rainbow scheme, from blue N-terminus to red C-terminus. PDB files of the corresponding models can be found in Supplementary data file 1.

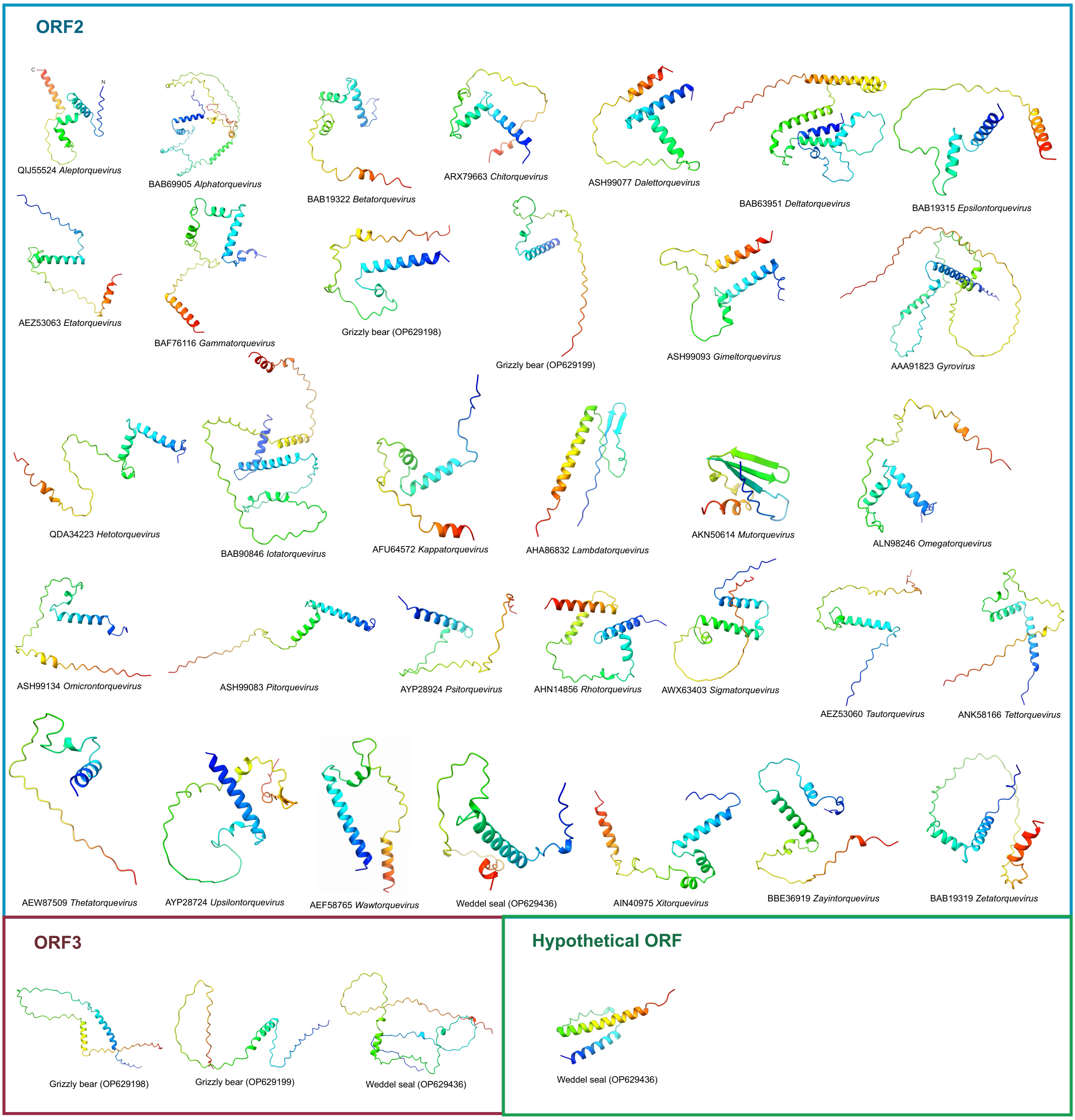

Supplementary Figure S3. The modelled anellovirus ORF2, ORF3 and hypothetical protein structures, one per genus, sorted alphabetically. The models are colored using the rainbow scheme, from blue N-terminus to red C-terminus.

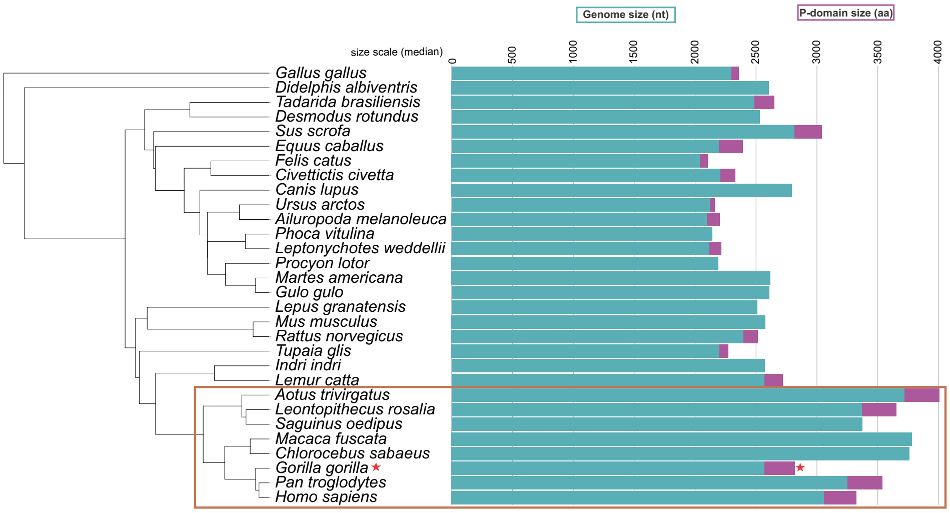

Supplementary Figure S4. The phylogenetic tree of anellovirus hosts with median values of genome and spike sizes shown for each host in a stacked bar plot. The green bars mark the genome sizes, while the purple bars mark the P-domain sizes and their lengths are proportional to the size of the bar. The spike sizes are shown for structures that were modelled. The yellow square marks the primates and their anelloviruses. The red star marks the gorilla viruses whose genomes are not complete.

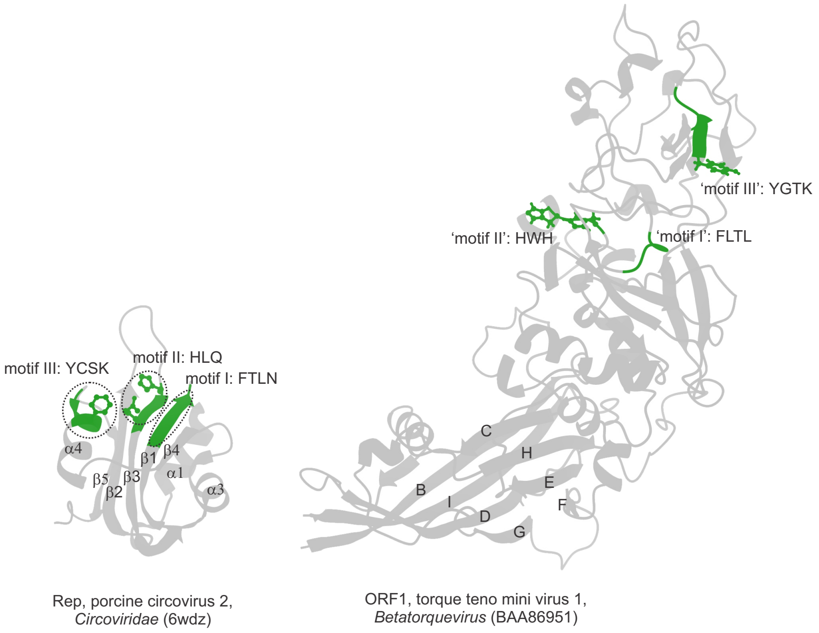

Supplementary Figure S5. Comparison of rolling-circle replication protein Rep from porcine circovirus (PDB:6WDZ) and the betatorquevirus ORF1 (torque teno mini virus 1). The green regions mark the distinct Rep motifs I-III on the Rep structure and the predicted "Rep motifs I-III" from the ORF1 alignment on the betatorquevirus structure.

Supplementary tables and data file legends

Supplementary table S1. Set of anellovirus-like primers used to recover complete genomes from each of the individual samples of the Weddell seals and grizzly bears.
(EXCEL)

Supplementary table S2. Anelloviruses used in the study from the VMR resource in ICTV. (EXCEL)

Supplementary table S3. Newly sequenced Weddell seal and grizzly bear sequences. (EXCEL)

Supplementary table S4. Results of the recombination analysis of lambatorquevirus sequences with newly sequences Weddell seal sequences using RDP5.
(EXCEL)

Supplementary table S5. Results of the HHsearch comparisons of anellovirus ORF1 sequences against the PDB70 and UniProt-SwissProt-viral70_3_Nov_2021 profile databases.
(EXCEL)

Supplementary table S6. Results of DALI structural comparisons of modelled anellovirus ORF1 structures against the PDB50 structural database.
(EXCEL)

Supplementary data file 1. Structural models of anellovirus ORF1 proteins generated using AlphaFold2.
(ZIP)
